# A Heuristic Framework for Image Filtering and Segmentation: Application to Blood Vessel Immunohistochemistry

**DOI:** 10.1155/2015/589158

**Published:** 2015-12-27

**Authors:** Chi-Hsuan Tsou, Yi-Chien Lu, Ang Yuan, Yeun-Chung Chang, Chung-Ming Chen

**Affiliations:** ^1^Institute of Biomedical Engineering, College of Medicine and College of Engineering, National Taiwan University, No. 1, Section 1, Jen-Ai Road, Taipei 100, Taiwan; ^2^Department of Radiology, National Taiwan University College of Medicine and Department of Medical Imaging, National Taiwan University Hospital, No. 7, Chung-Shan South Road, Taipei 100, Taiwan; ^3^Department of Internal Medicine, National Taiwan University College of Medicine, No. 7, Chung-Shan South Road, Taipei 100, Taiwan

## Abstract

The blood vessel density in a cancerous tissue sample may represent increased levels of tumor growth. However, identifying blood vessels in the histological (tissue) image is difficult and time-consuming and depends heavily on the observer's experience. To overcome this drawback, computer-aided image analysis frameworks have been investigated in order to boost object identification in histological images. We present a novel algorithm to automatically abstract the salient regions in blood vessel images. Experimental results show that the proposed framework is capable of deriving vessel boundaries that are comparable to those demarcated manually, even for vessel regions with weak contrast between the object boundaries and background clutter.

## 1. Introduction

Computer-aided diagnosis (CADx) for high-throughput tissue banks and digitized histological (tissue) images has shown promise for relieving pathologists' workload by assisting in differentiating cases of benign and difficult-to-diagnose suspicious tumor areas [1–5]. The CADx system is expected to improve clinical practice [1, 2] and the performance of human observers as they interpret histological images [3–5]. For example, the qualitative evaluation of the spatial distribution of vessels surrounding tumors may represent the increased levels of angiogenesis (the growth of new capillary blood vessels) in tumor growth [6–9]. To investigate the increased levels of tumorigenicity (the ability to give rise to tumors), the blood vessel density in a cancerous tissue sample can be determined by using immunohistochemical (IHC) staining methods. However, the interpretation of the histopathological image is relatively difficult and time-consuming, and the identification of blood vessels depends heavily on the observer's experience.

To overcome this drawback, computer-aided image analysis frameworks have been widely and intensively investigated in order to boost the performance of object identification in histological images [1]. Generally, a clinical decision support system starts with quality control and ends with predictive modeling for several cancer endpoints [10]. Nevertheless, it remains impractical to apply these computer-aided diagnostic algorithms in clinical applications. The primary difficulty lies in quantitatively characterizing the histological images, given the variety of imaging methods and disease-specific textures. Therefore, a pressing need exists for a computer-aided image analysis approach to quantify the useful factors for angiogenesis: area, spatial distribution, and density of blood vessels. Blood vessel region abstraction is a crucial step for achieving this goal. However, the development of robust algorithms for the automated analysis of blood vessel images has many challenges, including an extensive variation in blood vessel structure and features and missing blood vessel boundaries caused by weak contrast, background clutter, and stain contamination.

Based on the image analysis methods used for blood vessel quantification, our proposed framework includes two main components:

1. An image filtering algorithm that uses color, luminance, and spatial variations in pixel intensities in order to make a visual search more efficient.
2. An image segmentation algorithm that incorporates the global feature information derived from measurements of pixel intensities into the curve evolution and the curvature flow component.

One important advantage of using image filtering over a segmentation method is the ability to improve the accuracy of image segmentation; the operator can more easily distinguish the location of the vascular area. For instance, some variations in staining and scanning conditions, such as image acquisition protocols, capturing-device properties, and lighting conditions, can reduce the accuracy of the quantifications, rendering them unusable.

**Figure 1:**
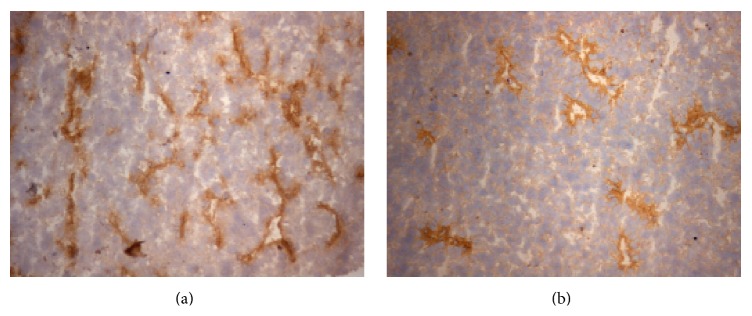
Immunostained vessel images.

Further, the structure and morphology of blood vessel images can be complicated, as shown in Figures 1(a) and 1(b). Traditional vessel image analysis methods [11–13] were unable to accurately detect the location of blood vessels. Therefore, we propose an image filtering algorithm allowing foreground (i.e., vessel) regions to be easily detected.

In this paper, we present a novel algorithm for automatically abstracting the blood vessel image that highlights the blood vessel regions and reduces the texture noise of the nonvessel regions. We examine the Gaussian color model of the original image to extract the large-scale layer and use the normalized color of the original color layer to extract the detailed layer. We then recombine the color layer of the original image, the large-scale layer, and the detailed layer to produce an image with two properties: salient blood vessel regions and a homogeneous background. Finally, we abstract this image using luminance quantization to generate the visually important blood vessel regions.

For vessel image quantification, the proposed image segmentation algorithm consists of two steps. In the first step, a region-based active contour method, namely, the graph partitioning active contours (GPAC) method, is used to derive a preliminary result. The GPAC incorporates global feature information into the curve evolution and the curvature flow component. Because misclassification is often inevitable in the GPAC segmentation of blood vessel images, owing to the weak contrast between the object boundaries and the background clutter, the second step further improves the segmentation accuracy using statistical intensity information. The essential concept of the second step is to employ local adaptive thresholding to discriminate between the subvessel regions formed in the first step and the background. It alleviates the interference caused by other structures within the subvessel regions, while preserving or introducing only tolerable distortion to the properties of the vessel objects of interest.

The remainder of this paper is organized as follows: We present the proposed framework in Section 2, discuss the results obtained from experiments in Section 3, and, finally, conclude the paper in Section 4. (Note: An earlier version of this work was presented as a conference paper [14]. This journal version extends the previous work with more concrete examples of complete theories, experiments, and comparisons.)

## 2. Materials and Methods

Three non-small cell lung-cancer cell lines, CL1-0 transfect, VEGF isoform 189, and A549, were used. All of them were cultured with the ATCC complete growth medium RPMI 1640, within a combination of 2 mM L-glutamine, 1.5 g/L sodium bicarbonate, 4.5 g/L glucose, 100 U/mL penicillin G sodium, 100 *μ*g/mL streptomycin sulfate, and 10% fetal bovine serum, in a humidified atmosphere consisting of 5% CO_2_ in air at 37°C. Immunohistochemical analysis of the cryostat sections and quantitative analysis of the blood vessel densities of tumor samples were performed. An anti-CD31 mouse monoclonal antibody (clone MEC 13.3, PharMingen) was used in the analysis. The proposed heuristic framework for histological vessel image analysis, based on the image filtering and segmentation algorithms, is detailed in the following sections.

### 2.1. Automatic Immunostained Vessel Image Filtering Algorithm

**Figure 2:**
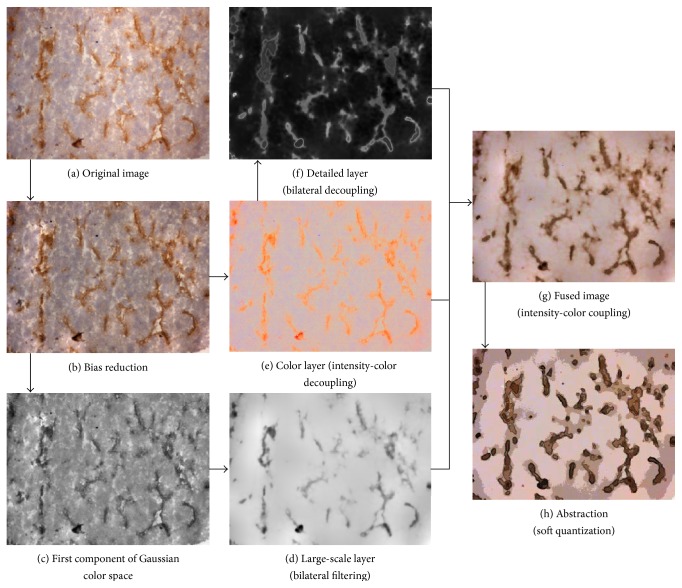
Flowchart of our proposed image filtering algorithm.

An algorithm for blood vessel abstraction [14] which was based on computing salient maps of blood vessel regions was proposed. Our approach proceeded as follows. First, we examined the stain adhered to the objects, using the normalized color on the color layer of the original image, to extract the detail of the blood vessel regions. This procedure could reduce the texture noise due to large structural variations in the biological image. Second, in order to maintain the pattern distributions, we used bilateral filtering to smooth the first component of the Gaussian color model and emphasize sharp features. Thus, we could preserve the blood vessel regions and simultaneously enhance their sharpness by reconstructing the detailed and large-scale layers. An overview of the complete algorithm is summarized in Figure 2.

Due to platform illumination variations in image acquisition, Niblack's adaptive thresholding method was performed to remove the light bias [15]. The basic concept of Niblack's method is to build a threshold surface *T*, based on the local mean *m* and standard deviation *s* of gray values, computed over a small neighborhood around each pixel in the form of

(1) $$\begin{array}{c} T=m+k\cdot s, \end{array}$$

where *k* is a negative constant. This method tends to produce the distribution of the light illumination. As a result, the light bias was reduced by dividing the intensity of the original by the threshold surface. The definition of the intensity and color channels is a linear weighted combination of *R*, *G*, and *B* for intensity estimation [16]:

(2) $$\begin{array}{c} I=\frac{R}{\left(R+G+B\right)}R+\frac{G}{\left(R+G+B\right)}G+\frac{B}{\left(R+G+B\right)}B. \end{array}$$

The intensity distribution of blood vessel regions was heterogeneous and the background of the original image was also cluttered. Therefore, using perceptual uniform color spaces in such images was necessary. We transformed the original RGB image into the Gaussian color space [17] using the following:

(3) $$\begin{array}{c} \left[\begin{array}{c} \hat{E}\\ {\hat{E}}_{\lambda }\\ {\hat{E}}_{\lambda \lambda } \end{array}\right]=\left(\begin{array}{ccc} 0.06 & 0.63 & 0.27\\ 0.3 & 0.04 & -0.35\\ 0.34 & -0.6 & 0.17 \end{array}\right)\left[\begin{array}{c} R\\ G\\ B \end{array}\right]. \end{array}$$

For human color vision, the first of three components $\hat{E}$,$\;\;{\hat{E}}_{\lambda }$, and ${\hat{E}}_{\lambda \lambda }$ of the Gaussian color model, measured by the Taylor expansion of the Gaussian weighted spectral energy distribution at *λ*_0_≃520 nm and scale *σ*_*λ*_≃55 nm, was used as an input for large-scale calculation. The invariant ${\hat{C}}_{\lambda }={\hat{E}}_{\lambda }/\hat{E}$ (the object's reflectance property, independent of viewpoint, surface orientation, illumination direction, or illumination intensity) was represented using normalized color to obtain the detailed layer of the original image.

After acquiring the normalized color, we used a bilateral decoupling procedure [16] to decompose the image into layers corresponding to the sharp details within the blood vessel regions. The bilateral filter [18] combined domain and range filtering by replacing the pixel value with a weighted average of similar (weight *r* on the pixel difference) and nearby (weight *s* on the spatial location) pixel values. The objective of the bilateral filter was to group perceptually similar colors together and preserve only the perceptually visible edge. Given an image *f*(·), the output *J*(·) of the bilateral filter for a pixel *x* was

(4) $$\begin{array}{c} J_{x}=\frac{1}{k\left(x\right)}\underset{n\in \Omega }{\sum }s\left(\;n-x\;\right)r\left(\;f_{n}-f_{x}\;\right)f_{n} \end{array}$$

with normalization

(5) $$\begin{array}{c} k\left(\;x\;\right)=\underset{n\in \Omega }{\sum }s\left(\;n-x\;\right)r\left(\;f_{n}-f_{x}\;\right). \end{array}$$

We used a combination of the bilateral filters of the normalized color to deduce the detailed layer. The large-scale layer was derived from a single iteration of a bilateral filter of the first component of the Gaussian color model. Then, we recombined the image using the element-by-element product of the color layer of the original image, the large-scale layer, and the detailed layer.

In order to abstract the blood vessel regions in the image obtained in the previous step, we modeled the visually salient regions by luminance quantization as follows [19]:

(6) Qs^,q,κq=qnearest+Δq2tanh⁡κq·hs^−qnearest,

where *Q*(·) is the pseudoquantized image, Δ*q* is the bin width, *q*_nearest_ is the bin boundary closest to $h(\hat{s})$, and *κ*_*q*_ is a parameter controlling the sharpness of the transition from one bin to another. The result of our approach is shown in Figures 2(h) and 9.

### 2.2. Histological Image Segmentation Algorithm

**Figure 3:**
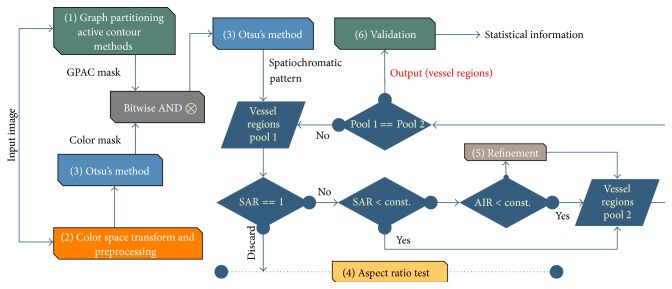
Overview of our proposed segmentation system.

The main stages of the proposed segmentation scheme are as follows: (1) the graph partitioning active contours (GPAC) method [20]; (2) color space transform and preprocessing; (3) Otsu's clustering method [21]; (4) aspect ratio test and refinement; and (5) validation.  Figure 3 shows the overview diagram of the proposed segmentation scheme.

#### 2.2.1. Graph Partitioning Active Contours Methods

The main objective of graph-based segmentation methods is to seek the best partition of the affinity graph, denoted as G, in which every image pixel is regarded as a graph node and every possible pairwise relation of image pixels is represented as a graph edge. The GPAC method [20] is a variational framework for pairwise-similarity-based segmentation which has an important characteristic called stability. This means it usually converges to the same result despite varying curve initializations and noise. Partitioning was used to derive the approximation of vessel regions in the image as an input of local adaptive thresholding while reducing computational efforts.

#### 2.2.2. Color Space Transform and Preprocessing

The intensity distribution of blood vessel regions was heterogeneous and the background of the original image was also cluttered. Therefore, using perceptual uniform color spaces in such images was necessary. As a result, we applied the transformation of the original RGB image into the YCbCr color space. Because of platform illumination variations in image acquisition, the process of image contrast enhancement using the sigmoid function in a spatial domain was used to correct the light bias.

#### 2.2.3. Threshold Selection

Otsu's method [21] is a bimodal clustering technique based on intensity estimation for the analysis of histogram distribution. Subvessel regions formed in the first step are discriminated from the background by minimizing the within-class variance of the regions formed by thresholding.

#### 2.2.4. Aspect Ratio Test and Refinement

**Figure 4:**
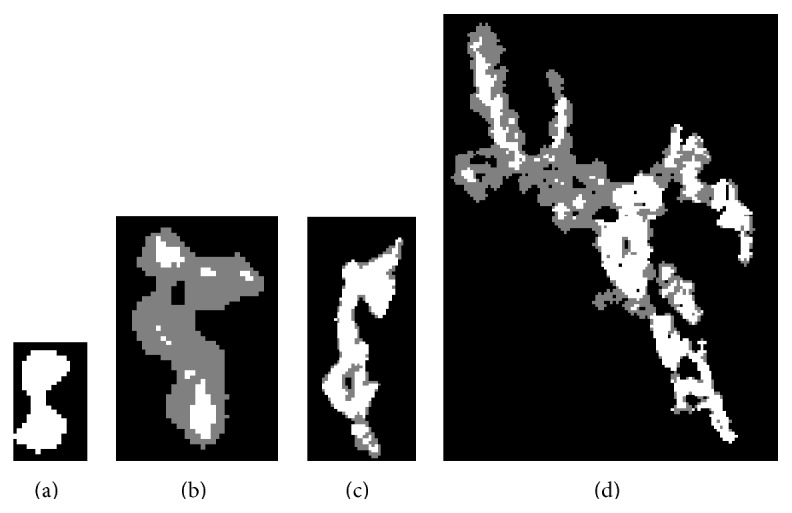
Four different spatiochromatic patterns. (a) Type 1; (b) Type 4; (c) Type 2; (d) Type 3.

Five different region types are derived, as shown in Table 1, according to their spatiochromatic patterns, as shown in Figure 4. The aspect ratio test comprises three criteria for distinguishing Type 3 regions from other type patterns. The definitions of these criteria are listed below.

**Criterion 1 .**

If the vessel-candidate region is Type 5 (i.e., all gray and no white tissue), then it will become part of the background (i.e., SAR = 1), thus eliminating unnecessary calculations.

**Criterion 2 .**

Calculate the surrounding area ratio (SAR) for each vessel-candidate region:

(7) $$\begin{array}{c} \text{SAR}=\frac{\text{surrounding tissue}}{\text{its vessel-candidate areas}}, \end{array}$$

where the surrounding tissue represents the gray part of the vessel-candidate area, which includes both gray and white areas.

**Criterion 3 .**

Calculate the area image ratio (AIR) for each vessel-candidate region:

(8) $$\begin{array}{c} \text{AIR}=\frac{\text{its vessel-candidate areas}}{\text{image size}}. \end{array}$$

**Figure 5:**
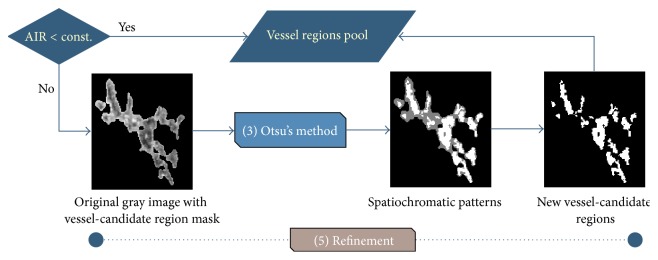
Refinement step process in the segmentation algorithm.

The aspect ratio test was conducted in order to identify the Type 3 pattern (i.e., half gray and half white). The relative vessel region is then arranged for the refinement step procedure, as shown in Figure 5.

#### 2.2.5. Validation

Our collaborating assistant manually calculated the vessel numbers in the images; we consider this to be the ground truth. We compared her numbers with the automatically generated numbers as follows:

(9) $$\begin{array}{c} \text{error}=\frac{Number\left(\text{computer XOR GT}\right)}{\text{Number}\left(GT\right)}\times 100\%, \end{array}$$

where computer is the region number (based on the automatically detected border) and ground truth (GT) was defined above.

## 3. Results and Discussion

### 3.1. Automatic Immunostained Vessel Image Filtering Algorithm

**Figure 6:**
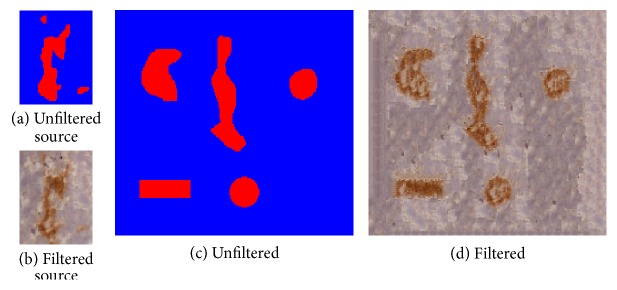
Synthetic vascular image. The unfiltered source image (a) was outlined manually to annotate (b). We created the unfiltered target image (c); then the filter learned from (a) and (b) was applied to (c) to get (d). The result is shown in (d).

**Figure 7:**
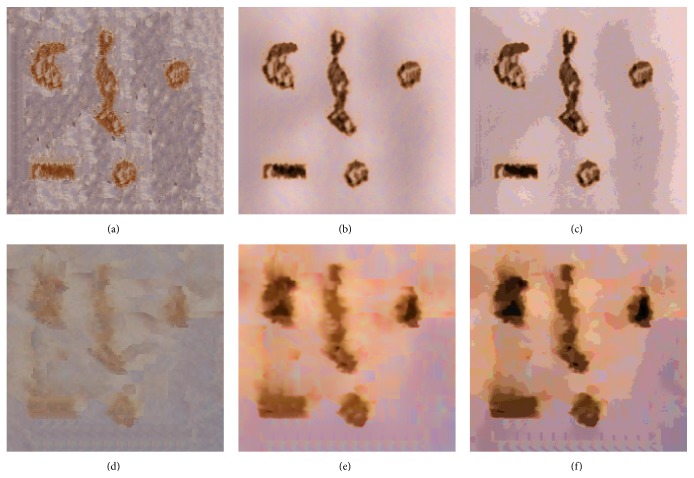
Synthetic vascular images. Abstraction results: original images (left), reconstructed images of the color layer of the original images, large-scale, and detailed layers (median), and salient blood vessel region abstraction from our proposed filtering method (right).

**Figure 8:**
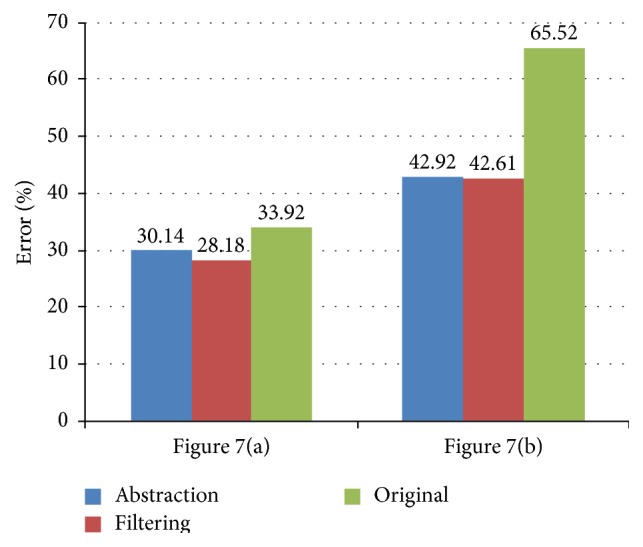
Error rates of image segmentation by fuzzy *c*-means clustering.

To create the synthetic vascular images with different complex vessel samples, we used “image analogies” [22] that effectively apply the statistics of a labeled image (Figure 6(a)) to a new unlabeled image (Figure 6(c)). Specifically, a synthetic vascular image (Figure 6(d)) was created by coating the given vessel samples (Figure 6(b)) with a labeling of the components of the vessel images. The experimental results of the synthetic vascular images in Figure 7 showed that filtering images within the nonvessel regions could improve the image segmentation accuracy using fuzzy *c*-means clustering, as shown in Figure 8, and preserve the vessel regions as well. We applied the three-class fuzzy *c*-means clustering to the synthetic vascular images and considered the first clustering as vessel pixels. The clustering error is defined as

(10) $$\begin{array}{c} \text{error}=\frac{\text{Area}\left(\text{computer XOR GT}\right)}{\text{Area}\left(\text{GT}\right)}\times 100\%, \end{array}$$

where computer is the binary image obtained by filling the image segmentation by fuzzy *c*-means clustering, and the ground truth (GT) is obtained from Figure 6(c).

**Figure 9:**
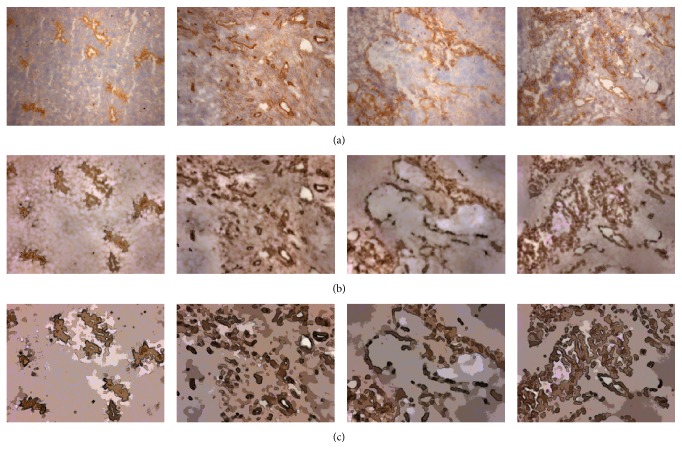
Abstraction results: original images (a); reconstructed images of the color layer of the original images, large-scale, and detailed layers (b); and salient blood vessel region abstraction from our proposed method (c).

**Figure 10:**
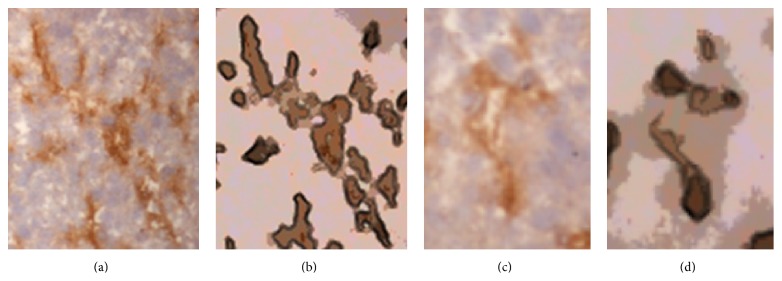
(a) Detail from Figure 2(a) with low contrast in the blood vessel regions. (b) Result of our proposed method with salient blood vessel regions. (c) Detail from Figure 2(a) with the cavity of the blood vessel surrounded by a few blood vessel wall areas. (d) Result of our proposed method with broken regions.

The experimental results of salient blood vessel region detection and abstraction using our method, shown in Figure 9, showed that the sharpness of the blood vessel borders was enhanced and the details of the blood vessel regions were also preserved. For instance, Figure 10(a) shows a detailed layer from Figure 2(a) with low contrast in the blood vessel regions while the background regions have high contrast and texture noise. The result of our proposed filtering method showed that our approach could increase the contrast in the blood vessel regions and also reduce the noise in the background regions, as shown in Figure 10(b).

In our experiment, if the margin of the blood vessel region was quite thin—for example, when the blood vessel cavity is surrounded by only a few blood vessel wall areas, as in Figure 10(c)—the final abstraction, as shown in Figure 10(d), would regard the cavity as background. This problem is currently under study, using machine learning techniques. In practice, we have gained promising abstraction results on a wide range of complex blood vessel images, as shown in Figure 9.

### 3.2. Histology Image Segmentation Algorithm

#### 3.2.1. Calculation Results

**Figure 11:**
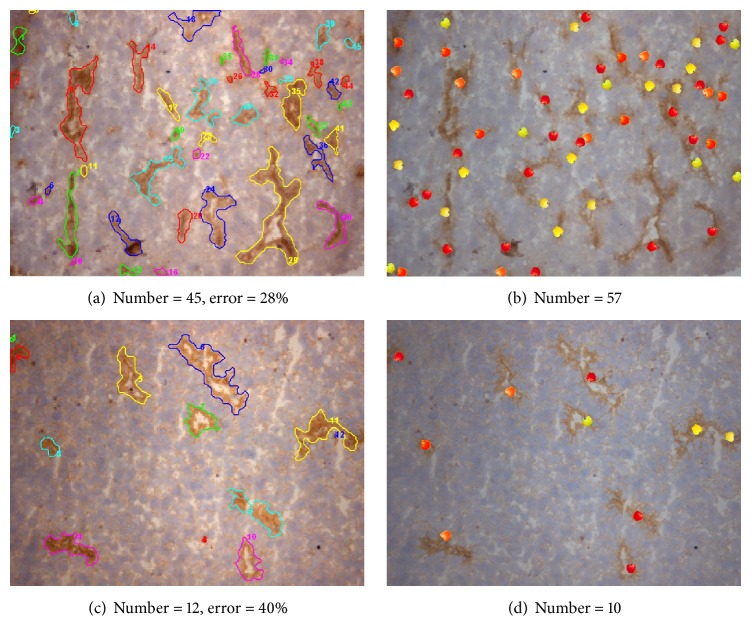
Calculation results. ((a), (c)) The boundaries are generated by our algorithm; ((b), (d)) those in apple dots are from the observer.

Figures 11(a) and 11(c) show the segmentation results. Figures 11(b) and 11(d) show the annotated locations of the blood vessels, as provided by the expert. It can be observed in Figure 11(a) that the blood vessel regions in the upper right with lower contrast are relatively difficult to identify, while the background regions have high contrast and texture noise. The result showed that our approach could delineate the major parts of the blood vessel regions and still do well in the blood vessel regions with the cavity, which is surrounded by few blood vessel wall areas.

#### 3.2.2. Comparison of Results

**Figure 12:**
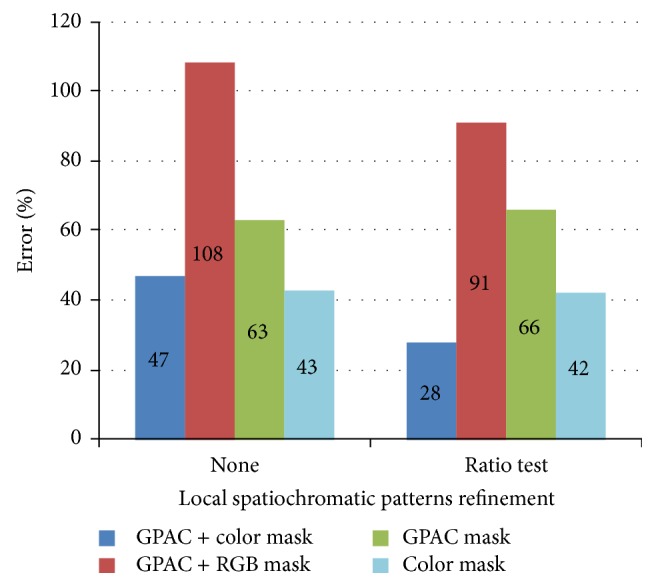
Comparison results.

Figure 12 compares the results of the proposed segmentation method with different spatiochromatic conditions. The results after applying the aspect ratio test were much better in some conditions in terms of the relationships between color style and vessel regions. Clearly, the proposed algorithm can better demarcate the blood vessel regions.

## 4. Conclusion

This paper provides a framework for automatically detecting and abstracting blood vessel regions using color, luminance, and other details from the original image. Currently, we are exploiting various unsupervised classifications to deal with the problem caused by a cavity in the blood vessel regions and are focusing on the implementation of a fully automated, objective, computer-based image analysis tool for the quantification of blood vessel images. We demonstrated that the implemented tool can calculate the vessel number and its area. The produced results were highly correlated with the human visual counts, conducted by an assistant from our department. Our next step is the exploration of various image textural features to achieve further quantification and automation in the assessment of vessel images.

## Figures and Tables

**Table 1:** Five different region types derived according to their spatiochromatic patterns.

	Type 1	Type 2	Type 3	Type 4	Type 5
Vessel (white)	All	Almost	Half	Few	None
Surrounding tissue (gray)	None	Few	Half	Almost	All
